# The deficiency in Th2-like Tfh cells affects the maturation and quality of HIV-specific B cell response in viremic infection

**DOI:** 10.3389/fimmu.2022.960120

**Published:** 2022-08-24

**Authors:** Alessandra Noto, Madeleine Suffiotti, Victor Joo, Antonio Mancarella, Francesco A. Procopio, Guy Cavet, Yvonne Leung, Jean-Marc Corpataux, Matthias Cavassini, Agostino Riva, Leonidas Stamatatos, Raphael Gottardo, Adrian B. McDermott, Richard A. Koup, Craig Fenwick, Matthieu Perreau, Giuseppe Pantaleo

**Affiliations:** ^1^ Service Immunology and Allergy, Lausanne University Hospital, University of Lausanne, Lausanne, Switzerland; ^2^ Atreca, Redwood City, CA, United States; ^3^ Service of Vascular Surgery, Lausanne University Hospital, University of Lausanne, Lausanne, Switzerland; ^4^ Service of Infectious Diseases, Lausanne University Hospital, University of Lausanne, Lausanne, Switzerland; ^5^ Division of Infectious Diseases, Luigi Sacco Hospital, University of Milan, Milan, Italy; ^6^ Department of Global Health, Seattle University of Washington, Seattle, WA, United States; ^7^ Vaccine and Infectious Disease Division, Fred Hutchinson Cancer Research Center, Seattle, WA, United States; ^8^ Vaccine Research Center, National Institute of Allergy and Infectious Diseases, National Institutes of Health, Bethesda, MD, United States; ^9^ Swiss Vaccine Research Institute, Lausanne University Hospital, University of Lausanne, Lausanne, Switzerland

**Keywords:** lymph nodes, follicular T helper cells, germinal center B cells (GC B cells), HIV-1 infection, T helper cell

## Abstract

Optimal T follicular helper (Tfh) cells function is important to promote the development of germinal centers and maturation of high affinity antigen-specific B cells. We have found that the expression of CXCR3 defines distinct Tfh subsets: CXCR3^+^ Th1-like Tfh cells mainly producing single IFN-γ and dual IL-21/IFN-γ and CXCR3^-^ Th2-like Tfh cells mainly producing single IL-4 and dual IL-21/IL-4 cytokines. CXCR3^-^ Th2-like Tfhs are significantly reduced during ongoing HIV replication. While the percentage of Th2-like Tfh cells correlates with that of total and cycling HIV-specific B cells, the percentage of CXCR3^+^ Th1-like Tfhs correlates with HIV-specific B cells expressing T-bet and CXCR3. Of note, only IL-4 and IL-21 cytokines boosted efficient maturation of HIV-specific B cells while IFN-γ induced expression of T-bet and CXCR3 in B cells. Interestingly, total and HIV-specific CXCR3^+^ B cells showed lower rate of somatic hypermutation, as compared to CXCR3^-^ B cells. Therefore, the imbalance in Th2/Th1-like Tfhs affects B cell responses in viremic HIV infection.

## Introduction

T follicular helper cells (Tfh) are a subset of memory CD4 T cells that represent a key component of protective immunity. Tfh are primarily found in the B-cell follicles of secondary lymphoid organs (SLO) where they interact with antigen-specific B cells providing efficient humoral immune responses. An efficient Tfh-B cell interaction is essential for the generation of high affinity and isotype class switched antibodies as well as for the establishment of long lasting memory B cells and plasma cells (1–5). Tfh cells are phenotypically defined by the coexpression of CXC Receptor 5, Program death-1 (PD-1), Inducible T-cell cell co-stimulator (ICOS) and by the expression of the B-cell lymphoma 6 protein (BCL-6) transcription factor, and functionally characterized by the production of IL-21 and IL-4 cytokines that together optimally drive B cell maturation (6–13)

Despite an increase in their frequency, Tfh cells from HIV-infected individuals are less effective at providing adequate B-cell help (14). The persistent antigenic stimulation drives the abnormal Tfh expansion and they remain capable of responding to HIV antigens but become functionally impaired (15, 16). Although multiple mechanisms might be involved in the functional impairment of Tfh cells, recent studies suggested a predominant role of increased expression of Programmed death-ligand 1 (PD-L1) in GC area (14).

Lymph node Tfh cells from simian immunodeficiency virus (SIV) infected animals and peripheral Tfhs from HIV-1 infected individuals have been shown to have a polarized T helper (Th)1-like phenotype and to express increased levels of T-bet and CXCR3 (17, 18). Futhermore, Th1-like Tfhs have increased production of Interferon (IFN)-γ and contain more copies of SIV DNA as compared to CXCR3^-^ Tfh cells in chronic SIV infection (17).

Of note, studies performed in Non-Human Primates (NHPs) and in humans have demonstrated that the frequency and the quality of Tfh cells specific to Env drives the magnitude and the quality of the Env-specific B cell response (19) and that they are required for the development of HIV broadly neutralizing antibodies (20). In this regard, an elegant study performed in mice has shown that the progressive differentiation of Tfh cells secreting IL-21 and IL-4 regulates the GC response (11).

Previous studies performed in tonsils and lymph nodes obtained from HIV-uninfected and/or HIV-infected viremic individuals have started to uncover the phenotypic heterogeneity of Tfh cells and highlighted certain phenotypic and functional (cytokines) differences between healthy and HIV infected viremic individuals (16, 21). Whether Tfh cells represent an heterogeneous population and whether different Tfh cell populations have a distinct impact on the generation of high affinity antibody response remains however unclear (13, 22–24).

In the present study, we have dissected the phenotypic and functional heterogeneity of Tfh cells in lymph nodes of HIV-infected ART treated and viremic individuals and in HIV-uninfected subjects, and determined their impact on the development of the Env-specific B cell response. We show that the expression of CXCR3 defines a Th1-like Tfh cell population functionally enriched in single IFN-γ and dual IFN-γ/IL-21 cells while CXCR3^-^ Th2-like Tfh cells are enriched in single IL-4 and dual IL-4/IL-21 producing cells. Of note, the Th2-like Tfh cell population was significantly reduced in HIV-infected viremic individuals. We provide evidence that the reduction in this Tfh cell population and the imbalance in the Th2 and Th1-like Tfh cell populations are associated with the phenotypic and functional abnormalities in the B cell responses of HIV viremic individuals. These results will be instrumental in developing strategies to optimize the induction of the magnitude and quality of antibody responses following vaccination.

## Results

### Characterization of Tfh cells

**Figure 1:**
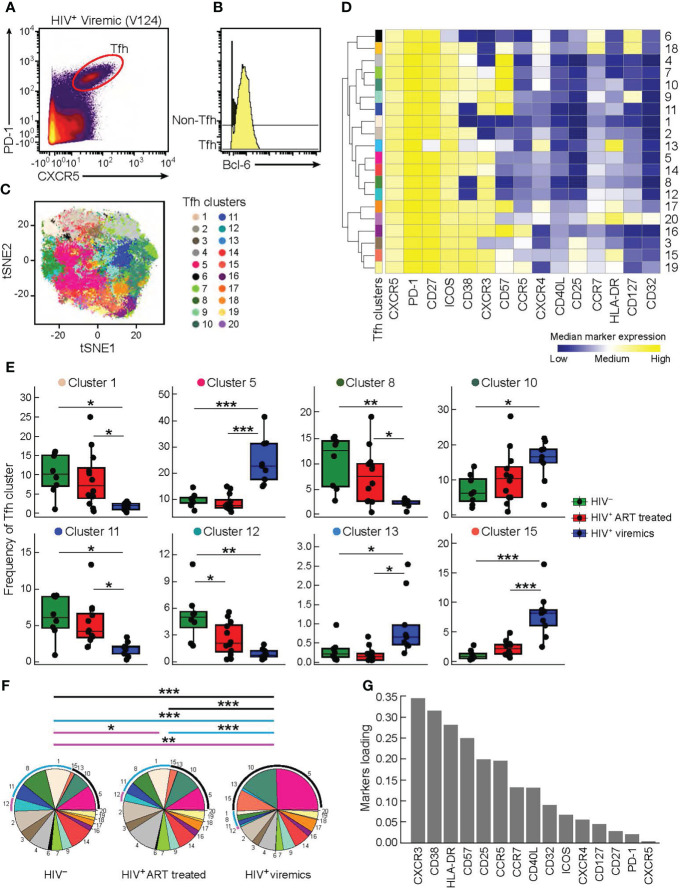
High dimensional analysis of Tfh cells in HIV infected and uninfected individuals. Mass cytometry staining was performed on LN mononuclear cells isolated from 8 HIV uninfected, 12 HIV infected HIV-infected ART treated individuals, and 9 viremic individuals. **(A)** Representative Tfh mass cytometry gating strategy on the basis of CXCR5 and PD-1 expression in CD45RA^-^CD4^+^ cells from representative viremic HIV infected individual and **(B)** BCL-6 expression on Tfh and non-Tfh cells. **(C)** t-SNE was performed after pooling the three study groups and gating on Tfh cells. The numbered and colored clusters Tfh clusters were obtained using FlowSOM. **(D)** Heat map showing median marker expression (arcsinh-transformed) of cell surface markers of the indicated clusters identified in **(C)**. Median marker expression values are color-coded from blue (low) to yellow (high) and the branching on the left of the figure represent how related the populations are according to the marker expression. The height of the branches are proportional to the distance. **(E)** Frequency of Tfh clusters that are significantly different between the three groups (HIV uninfected (green), HIV-infected ART treated individuals (red) and viremic (blue) individuals). **(F)** Pie charts representing frequencies of Tfh clusters in HIV uninfected, HIV-infected ART treated individuals and HIV-infected viremic individuals. Arcs show frequencies of clusters that are significantly different between HIV uninfected, HIV-infected ART treated individuals and HIV-infected viremic individuals. **(G)** Bar plot showing the relative contribution of markers to Tfh clusters heterogeneity. (Y-axis: average marker loadings in the first two principal components of a PCA). *P* values were obtained by linear regressions and corrected using FDR method with a cutoff of 0.05. *P < 0.05, **P < 0.01, ***P < 0.001.

In order to dissect the heterogeneity of Tfh cells, lymph node biopsies were obtained from 8 HIV-uninfected individuals, 12 HIV-infected aviremic ART treated individuals and 9 HIV-infected viremic individuals (**Table 1**). Notably, the age and time post infection were significantly different between ART treated and untreated viremic individuals (median age: 47 years *versus* 36 years; median duration of HIV infection: 10.86 years *versus* 0.18 years, respectively), while no significant differences were observed in terms of gender (*P*>0.05). Of note, the gender and age parameters of HIV-uninfected individuals were not collected in the present sudy. Lymph node mononuclear cells (LNMCs) were then characterized with a unique panel of 30 markers of T cell activation, memory differentiation, chemokine receptors and HIV coreceptors (**Supplementary Table 1A**). Tfh cells were defined by gating on memory CD4 T cells on the basis of the expression of PD-1 and CXCR5 and high levels of BCL-6 (**Supplementary Figure 1** and **Figures 1A, B**). Unsupervised clustering was performed on pooled Tfh cells from the three study groups HIV-infected aviremic, ART treated individuals and HIV-infected viremic individuals by a data-driven unsupervised clustering method, i.e., FlowSOM, in combination with consensus clustering. This analysis defined 20 different populations (i.e. clusters) within CXCR5^high^PD-1^high^ Tfhs. T-distributed stochastic neighbor embedding (t-SNE) was used to perform dimensions reduction and visualize our data in a two-dimensional plot that placed cells with similar phenotypic characteristics (in high dimensional space) in close proximity (**Figure 1C** and **Supplementary Figures 2A, B**). After clusters definition, we used the heat map (**Figure 1D**) to show the phenotypic profile of each cluster in terms of median marker intensity for an individual marker. The 20 defined clusters showed variations in the expression of CD38, CXCR3, CD57, HLA-DR, CD127, CXCR4, and to less extend of CCR5, CD25, CCR7 and CD32 while Tfhs were homogeneous for the expression of CD27, ICOS and CD40L (**Figure 1D**).

**Table 1:** Study cohort: clinical data.

Patient ID	Age	Sex	Duration of HIV Infection (years)	Viral Load (copies/ml)	CD4 count (cells/ul)	ART status
T001	49	M	25.23	<20	549	ART Treated
T006	53	M	9	<20	648	ART Treated
T008	53	M	7.78	<20	NA	ART Treated
T010	54	F	15.76	<20	703	ART Treated
T011	57	M	20.1	<20	854	ART Treated
T042	58	M	11.1	<20	635	ART Treated
T058	46	F	10.86	<20	666	ART Treated
T060	42	M	21	<20	786	ART Treated
T061	43	M	23	<20	698	ART Treated
T062	47	M	5.12	<20	487	ART Treated
T067	41	M	14.01	<20	609	ART Treated
T068	45	M	3.33	<20	928	ART Treated
T069	47	M	5.12	<20	487	ART Treated
T070	55	M	7.06	<20	615	ART Treated
T072	47	M	18	<20	614	ART Treated
T078	38	M	6.27	<20	728	ART Treated
T081	51	M	22.45	<20	1236	ART Treated
T082	52	M	23.49	<20	1093	ART Treated
T083	54	F	23.27	<20	941	ART Treated
T093	41	F	3	<20	336	ART Treated
T094	47	M	8	<20	451	ART Treated
T096	54	F	18	<20	1253	ART Treated
T100	36	F	13.06	<20	643	ART Treated
T103	41	M	1.62	<20	217	ART Treated
T104	41	F	10	<20	455	ART Treated
T108	50	M	11	<20	490	ART Treated
T123	61	M	9.25	<20	1270	ART Treated
T136	44	M	7.5	<20	732	ART Treated
T137	39	M	3.59	<20	700	ART Treated
V005	38	M	8	5186	651	Treatment Naive
V015	25	M	0.14	17000	416	Treatment Naive
V017	34	M	0.2	32000	602	Treatment Naive
V018	48	M	2	260000	475	Treatment Naive
V019	NA	F	NA	73400	634	Treatment Naive
V031	32	F	0.2	90000	819	Treatment Naive
V034	24	M	0.3	5000	549	Treatment Naive
V035	34	M	6.1	3000	517	Treatment Naive
V037	37	M	0.3	25000	704	Treatment Naive
V038	38	M	0.1	1500000	550	Treatment Naive
V049	45	M	0.1	20000	704	Treatment Naive
V056	35	F	13	830	670	Treatment Naive
V102	36	M	NA	83000	602	Treatment Naive
V106	37	M	0.1	160000	501	Treatment Naive
V117	51	M	5.21	54000	504	Treatment Naive
V119	23	M	1.3	13000	498	Treatment Naive
V118	29	M	0.06	14000	538	Treatment Naive
V124	46	F	0.06	510000	468	Treatment Naive
V125	35	M	0.16	17000	511	Treatment Naive
V140	23	M	0.08	360000	427	Treatment Naive
V143	31	M	0.1	10000000	174	Treatment Naive
V148	38	M	0.06	20000	717	Treatment Naive
V149	39	M	0.1	18000	280	Treatment Naive
V150	53	M	10	6900	578	Treatment Naive

NA, not applicable.

Next, we found that only 8 out of the 20 Tfh cell clusters were significantly differentially distributed in the three study groups (**Figure 1E** and **Supplementary Figure 2C**). Clusters 1, 8 and 11 were significantly decreased in viremics as compared to ART treated and healthy individuals. These three clusters accounted for the 29.3% and 26.84% of the total Tfh cell population in ART treated and healthy individuals, respectively, and in viremics only 5.9% (*P* < 0.0001) (**Figure 1F**).

Clusters 5, 10, 13 and 15 were over represented in viremics and accounted for 49.17% of the total Tfh cell population (cluster 5: 24.4%; cluster 10: 15.53%; cluster 13: 0.89%; cluster 15: 8.35%), while in ART treated and HIV-uninfected individuals they accounted for 22.14% and 18.66%, respectively (*P* < 0.0001) (**Figure 1F**).

Only cluster 12 was significantly increased in HIV-uninfected individuals as compared to HIV-infected ART treated (*P* = 0.048) and viremic individuals (*P* = 0.0014) (on average HIV-uninfected: 5.12%; HIV-infected ART treated individuals: 2.63%; HIV-infected viremic individuals: 0.89%) (**Figures 1E, F**).

Clusters with higher frequencies in HIV-infected viremic individuals co-expressed CXCR3 and CD38 with varying levels of CD57, CXCR4, HLA-DR and CD25 (**Figures 1D**) while those abundant in HIV-infected ART treated individuals and HIV-uninfected individuals expressed lower levels of CD38 and CCR5 and were heterogeneous in CXCR3 expression (**Figure 1D**). This was further confirmed by unsupervised principal component analysis (PCA) which showed that the markers that contributed the most to Tfh heterogeneity were CXCR3 and CD38 (**Figure 1G**).

Alltogether these data highlight the phenotypic heterogeneity of Tfhs and define distinct phenotypic subsets of Tfhs that are differentially distributed in HIV-uninfected, HIV-infected ART treated individuals and viremic individuals.

### Relationship between CD38 and CXCR3 Tfh cell populations

**Figure 2:**
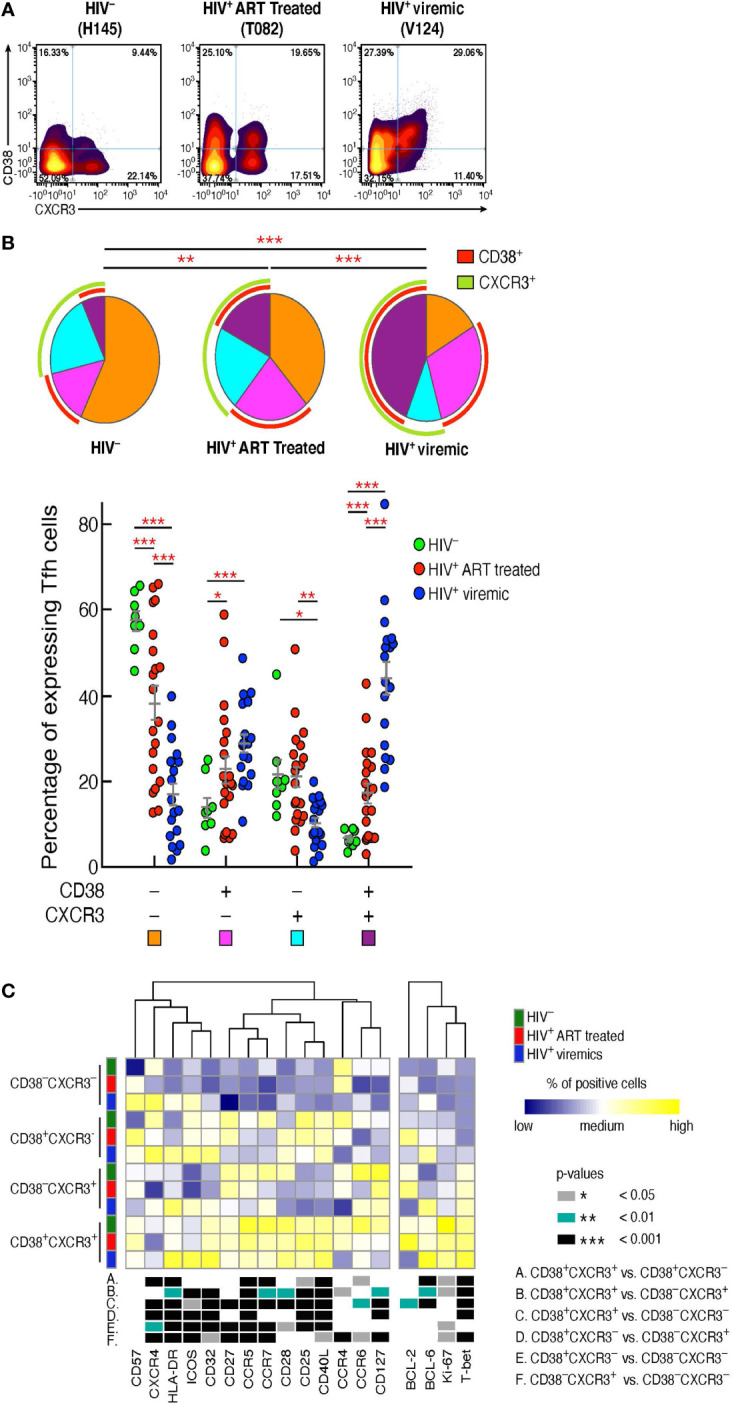
CD38^+^CXCR3^+^ Tfh cells are increased in HIV-infected viremic individuals and represent a distinct Tfh population. Mass cytometry staining was performed on LN mononuclear cells isolated from 8 HIV uninfected, 20 HIV infected HIV-infected ART treated individuals and 18 untreated HIV-infected viremic subjects. Cells were stained with antibodies against PD-1, CXCR5, CD38, CXCR3. **(A)** Representative profile by mass cytometry of Tfh cells gated on the basis of CD38 and CXCR3 from one representative HIV uninfected, one HIV-infected ART treated individuals and one viremic individual. **(B)** All the possible combinations of CD38 and CXCR3 expression are shown on the x axis, whereas the frequencies of the Tfh-cell populations are shown on the y axis. Pie charts represent all the possible combinations of the two markers. Arcs show the total proportion of the expression of the specified marker. Statistical analyses of the global CD38 and CXCR3 expression (pie charts) were performed by partial permutation tests using the SPICE software. **(C)** Heat map of scaled mean marker expression (percentage of positive cells) in Tfh cells defined on the basis of CD38 and CXCR3 expression of 8 HIV uninfected, 12 HIV-infected ART treated individuals and 9 viremic individuals. The bottom panel shows significant differences between subsets of cells for all possible comparisons. Differences between subsets were calculated on all cohort samples using linear mixed-effect models. In **(C)** *P* values were obtained by linear regressions and corrected using FDR method with a cutoff of 0.05. Stars indicate statistical significance *P < 0.05, **P < 0.01, ***P < 0.001.

Having delineated the phenotypic markers defining the Tfh cell populations differentially distributed in the three study groups, we sought to confirm the findings generated by FlowSOM in combination with consensus clustering by manual gating. The additional analyses confirmed that Tfh cells co-expressing CD38 and CXCR3 were consistently increased in HIV-infected viremic individuals as compared to HIV-uninfected (44.1% vs 6.7%, *P* < 0.0001) and HIV-infected ART treated individuals (17.4%, *P* < 0.0001) (**Figures 2A, B**). CD38^-^CXCR3^+^ cells were significantly reduced in HIV-infected viremic individuals as compared to HIV-infected ART treated (10.5% vs 21.19%, *P* = 0.001) and to HIV-uninfected subjects (20%, *P* = 0.017) (**Figures 2A, B**) while CD38^+^CXCR3^-^ cells were significantly increased in HIV-infected viremic individuals as compared to HIV-uninfected subjects (28.9% vs 14.11%, *P* < 0.0001) but not as compared to HIV-infected ART treated subjects (22.9%, *P* = 0.14) (**Figures 2A, B**).

We next determined the levels of expression of markers associated with memory cell differentiation, cell activation, and cell trafficking within the Tfh cell populations defined by the expression of CD38 and CXCR3 in the three study groups (**Figure 2C**). The heat map shows that the large majority of markers defining T cell activation (Ki-67, CD25, HLA-DR) were significantly increased in CD38^+^CXCR3^+^ Tfhs as compared to the other Tfh cell populations. Of note, the expression of the HIV coreceptor CCR5 was greatly increased within CD38^+^CXCR3^+^ Tfhs (*P* < 0.0001) in the three study groups (67% in HIV-uninfected, 55.8% HIV-infected ART treated individuals and 44.5% in HIV-infected viremic individuals) (**Figure 2C**), suggesting that this subset might potentially be more susceptible to HIV infection.

The Th1 cell lineage T-box transcription factor (T-bet) was significantly increased in CD38^+^CXCR3^+^ Tfhs as compared to CD38^-^CXCR3^+^, CD38^+^CXCR3^-^ and CD38^-^CXCR3^-^ Tfhs (*P* < 0.001), while the Th2 transcription factor GATA-3 was significantly increased in CD38^-^CXCR3^-^ Tfh cells (**Figure 2C**) (*P* < 0.01). Of note, GATA-3 expression within the CD38^-^CXCR3^-^ Tfh cells was significantly decreased in HIV infected viremic individuals as compared to HIV-infected ART treated subjects (0.81% in HIV-infected viremic individuals vs 30.8% in HIV-infected ART treated individuals, *P* < 0.0001) and HIV uninfected individuals (12.04%, *P* = 0.0006). Similarly, the Th2 specific chemokine, CCR4, was strongly reduced in all Tfh cells of HIV-infected viremic individuals as compared to both HIV-uninfected and HIV-infected ART treated subjects (1.6 to 2 fold, *P* < 0.01).

Taken together, these results indicate that there is an association between the CD38 and CXCR3 expansion profile and the Tfh-cell polarization; in particular, CD38^+^CXCR3^+^ cells identify a population of activated and Th1 polarized Tfh cells, while Th2 polarized Tfh cells were CD38^-^CXCR3^-^ and significantly reduced in viremic individuals.

### Functional characterization of CD38^+^CXCR3^+^ Tfh cells

**Figure 3:**
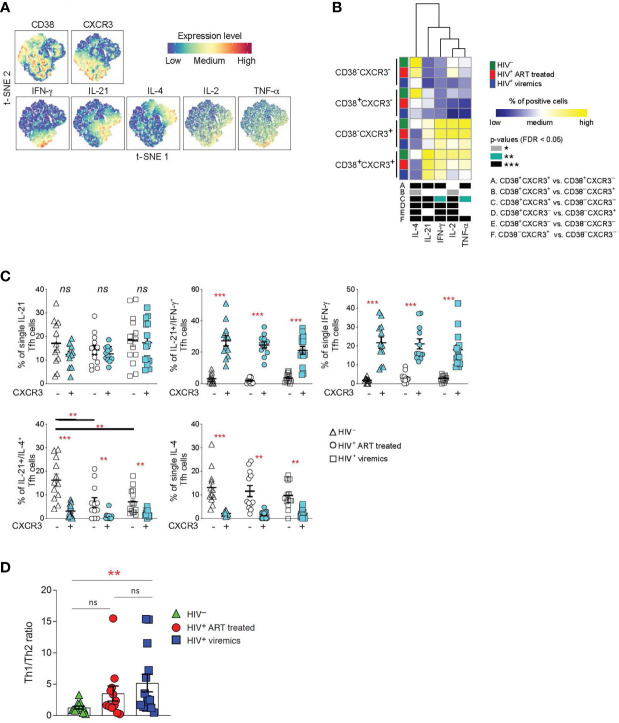
Functional analyses of Tfh cells on the basis of CD38^+^ and CXCR3^+^ expression. LNMCs were isolated from HIV uninfected (N = 12), HIV-1 infected HIV-infected ART treated individuals individuals (N = 12) and HIV-infected viremic individuals (N = 14) and stimulated with PMA-ionomycin for 5 hours and stained with antibodies against PD-1, CXCR5, CD38, CXCR3, IFN-γ, IL-21, IL-4, IL-2 and TNF-α. **(A)** t-SNE plot of pooled Tfh cells from the three study groups (51’171 cells). **(B)** Heat map of scaled mean marker expression (percentage of producing cytokines) in Tfh cells gated on the basis of CD38 and CXCR3. The bottom panel shows significant differences between subsets of cells for all possible comparisons. **(C)** Simultaneous analysis of the functional profile of CXCR3^-^ and CXCR3^+^ Tfh cells on the basis of IL-21, IL-4 and IFN-γ production. **(D)** Th1/Th2 ratio in Tfh cells from HIV uninfected, HIV infected viremic and HIV-infected ART treated individuals individuals. Ratio was calculated by dividing the frequencies of IFN-γ producing Tfh cells and the frequencies of IL-4 producing Tfh cells in HIV- and HIV HIV-infected viremic individuals. In **(B)** differences between subsets were calculated on all cohort samples using linear mixed-effect models and *P* values were corrected using FDR method with a cutoff of 0.05. In **(C, D)** *P* values were obtained by a Mann-Whitney test to compare the three study groups and a Wilcoxon signed-rank test to compare frequencies between CXCR3^-^ and CXCR3^+^ populations. *P < 0.05, **P < 0.01, ***P < 0.001. Error bars denote mean ± S.E.M. ns, not significant.

Tfh cells produce high levels of IL-21, a cytokine that is critical for GC formation and B cell maturation (25, 26). Although IL-21 is the signature cytokine of Tfh cells, studies have shown that Tfh cells are also able to produce cytokines typical of other cell lineages of helper CD4 T cells (13). Therefore, we determined the cytokine profile of Tfh cells on the basis of CXCR3 and CD38 expression. LNMCs from HIV-uninfected, HIV-infected ART treated and HIV-infected viremic individuals were stimulated for 5 hours in the presence of PMA and ionomycin and the cytokine profile was evaluated by mass cytometry.

t-SNE analysis showed a dichotomy in the distribution of the Th1 IFN-γ and Th2 IL-4 cytokinesbetween CXCR3^+^ and CXCR3^-^ Tfh cell populations whereas the two cytokines were spread within CD38^+^ and CD38^-^ Tfh cells (**Figure 3A**). The dichotomy in the distribution of IFN-γ and IL-4 between CXCR3^+^ and CXCR3^-^ Tfh cell populations was further confirmed in the heat map (**Figure 3B**). CD38^-^CXCR3^-^ and CD38^+^CXCR3^-^ Tfh cells on one side and CD38^-^CXCR3^+^ and CD38^+^CXCR3^+^ on the other side share similar functional cytokine profiles and are closely related to each other. CXCR3^-^CD38^-^ and CXCR3^-^CD38^+^ Tfh cells were predominantly enriched in IL-4 whereas CXCR3^+^CD38^-^ and CXCR3^+^CD38^+^ Tfh cells were significantly enriched in IFN-γ and also in IL-21, IL-2 and TNF-α.

Taken together these results demonstrate that the expression or the lack of CXCR3 defines two functionally distinct Tfh cell subsets: CXCR3^+^ Tfh/Th1-like IFN-γ^+^ and CXCR3^-^ Tfh/Th2-like IL-4^+^.

We therefore analyzed the distribution of Th1 and Th2 cytokines within the CXCR3^+^ and CXCR3^-^ cell populations in the three study groups. Interestingly, we identified a population of single IL-4 and dual IL-21/IL-4 cytokine producing Tfh cells within the CXCR3^-^ Th2-like Tfh cells; while single IFN-γ and the dual IL-21/IFN-γ producing Tfh cells were enriched within the CXCR3^+^ Th1-like Tfh cell populations (**Figure 3C**). Interestingly, the percentage of dual IL-21/IL-4, and to a lesser extent of single IL-4 Tfh, was significantly reduced in HIV-infected viremic individuals as compared to HIV-uninfected subjects (**Figure 3C**). Of note, the Th1-like/Th2-like ratio, calculated by the ratio between the frequency of total IFN-γ^+^ Tfh cells and the frequency of total IL-4 producing Tfh cells, was significantly increased in HIV-infected viremic individuals as compared to HIV-uninfected subjects (5.1 vs 1.2, *P* = 0.003) (**Figure 3D**). Furthermore, treatment with ART showed a trend towards the recovery of Th2-like Tfh cells (**Figure 3D**).

Therefore, these results indicate that Tfh cells from HIV infected individuals are skewed towards a CXCR3^+^ Th1-like-Tfh population.

### Frequency and distribution of gp140-specific B cells in HIV-infected ART treated and HIV-infected viremic individuals

**Figure 4:**
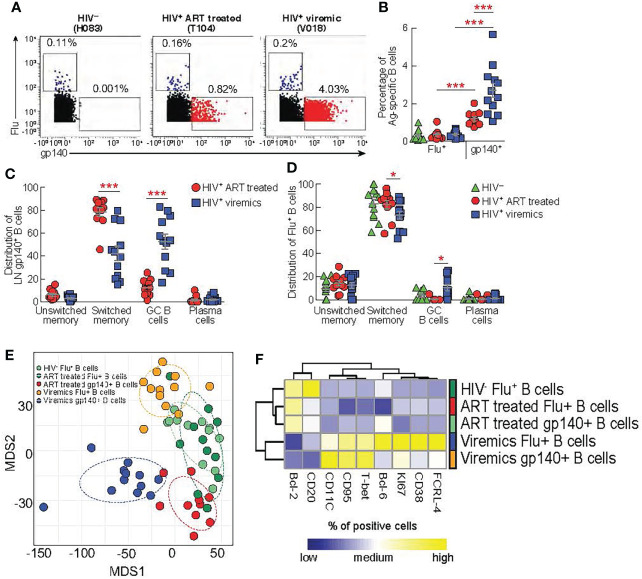
Comparison between lymph node gp140 and influenza specific B cells. LNMCs from HIV uninfected (N = 12) infected HIV-infected ART treated individuals (N = 11) and HIV infected viremic individuals (N = 12) were stained with a panel of 37 markers (**Table S1B**). **(A)** Representative mass cytometry profiles of LN CD19^+^ IgG^+^ B cell populations binding to gp140 or Flu (H1-CA09) probes in representative HIV^-^, HIV-infected ART treated individuals and viremic subjects. **(B)** Cumulative data on the frequencies of Flu and gp140-specific B cells in LNs of HIV^-^ (green), HIV-infected ART treated individuals (red) and viremic (blue) individuals. Cumulative data on the distribution of **(C)** gp140-specific B cells and **(D)** Flu-specific B cells within the unswitched memory (IgD^+^CD38^-^), switched memory (IgD^-^CD38^-^), GC (IgD^-^CD38^+^) and plasma cells (IgD^-^CD38^hi^) B cell populations. **(E)** Multi-dimentional scaling (MDS) of Flu^+^ B cells, gp140^+^ B cells from HIV^-^, ART-treated and viremic HIV-infected subjects. **(F)** Heat map of scaled mean marker expression (% of positive cell gated) in gp140-specific B cells from HIV-infected ART treated individuals and viremic individuals, and Flu-specific from HIV uninfected HIV-infected ART treated individuals and viremic individuals. All markers shown are significantly different between groups (FDR < 0.05). Differences were calculated using linear regressions. In **(B‐D)** *P* values were obtained by Mann-Whitney test to compare frequencies between the three study groups and by Wilcoxon signed-rank test to compare frequencies within the same study groups. Error bars denote mean ± S.E.M. Stars indicate statistical significance *P < 0.05, ***P < 0.001.

We have previously shown that the acquisition of optimal Tfh cell functions is important to deliver adequate help to GC B cells for the development of functional antigen-specific B cells producing potent neutralizing antibodies (12, 13). Our results indicate that CXCR3^-^ Th2-like Tfh cells, potentially important for promoting efficient B cell maturation, are quantitatively reduced in viremic individuals. Therefore, we firstly investigated the quality and quantity of LN antigen-specific B cells and secondly we assessed the relationship between skewed Th1-like Tfh cells and antigen-specific B cell responses. We have taken advantage of the use of a biotinylated gp140 trimer linked to metal conjugated streptavidin in order to identify HIV-specific B cells from lymph nodes by mass cytometry (27). Notably, Influenza (Flu^+^) -specific B cells were used as control of B cell responses within the same lymph nodes of HIV-uninfected, HIV-infected ART treated and viremic individuals in flu vaccinated individuals. After gating on CD19^+^ cells to identify B cells and on IgG^+^ cells to enrich in memory B cells, gp140 trimers and HA protein were used to identify HIV-specific and Flu-specific B cells, respectively (**Figure 4A**). The frequency of LN gp140-specific B cells was significantly higher in HIV-infected viremic individuals versus HIV-infected ART treated individuals (2.7% vs 1.2%, *P* = 0.0005) (**Figures 4A, B**) and in LNs as compared to peripheral blood (HIV-infected viremic individuals: 2.7% in LNs vs 1% in blood, *P* = 0.006; HIV-infected ART treated individuals: 1.2% in LNs vs 0.5% in blood, *P* = 0.002; HIV-uninfected: 0.001%) (**Supplementary Figures 3A, B**). Flu-specific B cells were detected at similar frequencies in LNs from the three study groups (about 0.2% of IgG^+^ B cells) but at significantly lower frequencies as compared to the percentage of gp140-specific B cells in HIV-infected ART treated and viremic individuals (*P* = 0.001 and *P* = 0.002, respectively) (**Figures 4A, B**). Next, we determined the distribution of gp140- and Flu-specific B cells within memory B cell subsets using mass cytometry. In LNs, IgD and CD38 identify four populations of non-naive B cells: unswitched memory IgD^+^CD38^-^, switched memory IgD^-^CD38^-^, GC B cells IgD^-^CD38^+^ and plasma cells IgD^-^CD38^hi^ (13). After gating on non-naive B cells (naive IgD^+^CD27^-^ (<1%) were excluded) the phenotypic analysis showed that gp140-specific B cells in LNs from viremic individuals were primarily contained within GC B cells (52.6%) and switched memory (42.3%), while gp140-specific B cells from HIV-infected ART treated individuals were mostly contained within the switched memory B cell population (78.3%) (**Supplementary Figure 4** and **Figure 4C**). Flu-specific B cells were mostly contained within switched memory B cells in the three study groups (around 80%) (**Figure 4D**).

Next, we performed multivariate analysis using multi-dimensional scaling (MDS) to examine the general distribution of antigen specific B cells. The MDS plot clearly showed that gp140-specific B cells from HIV-infected ART treated and viremic individuals clustered separately and away from Flu^+^ B cells within the same HIV-infected individuals. Interestingly, Flu^+^ B cells from HIV-infected ART treated individuals and HIV-uninfected individuals partially overlapped while Flu^+^ B cells from HIV-infected viremic individuals clustered away from these two groups (**Figure 4E**). Next, we analyzed the expression of B cell markers (**Supplementary Table 1B**) in the Ag-specific B cells from the three study groups. Similarly to the MDS analysis, we found that HIV-specific and Flu-specific B cells from HIV-infected viremic individuals shared a similar phenotype (high levels of T-bet, CD95, KI-67, CD11c, FCRL-4 and lower levels of CD20 and BCL-2) (**Figure 4F**). Notably, HIV-specific B cells from HIV-infected ART treated individuals and Flu-specific B cells from both HIV-infected ART treated individuals and HIV-uninfected individuals expressed reduced expression of markers associated with inductive or effector stages (low levels of T-bet, Ki67, CD38, FCRL-4, CD95 and CD11c)(**Figure 4F**).

Overall, these results indicate that the ongoing HIV replication shapes the profile of HIV-specific B cells and that the ongoing HIV replication in LNs is associated with changes in the phenotypic profile of Ag-specific B cells that are not restricted exclusively to HIV-specific B cells.

### Relationship between Tfh cell populations and gp140-specific B cells

**Figure 5:**
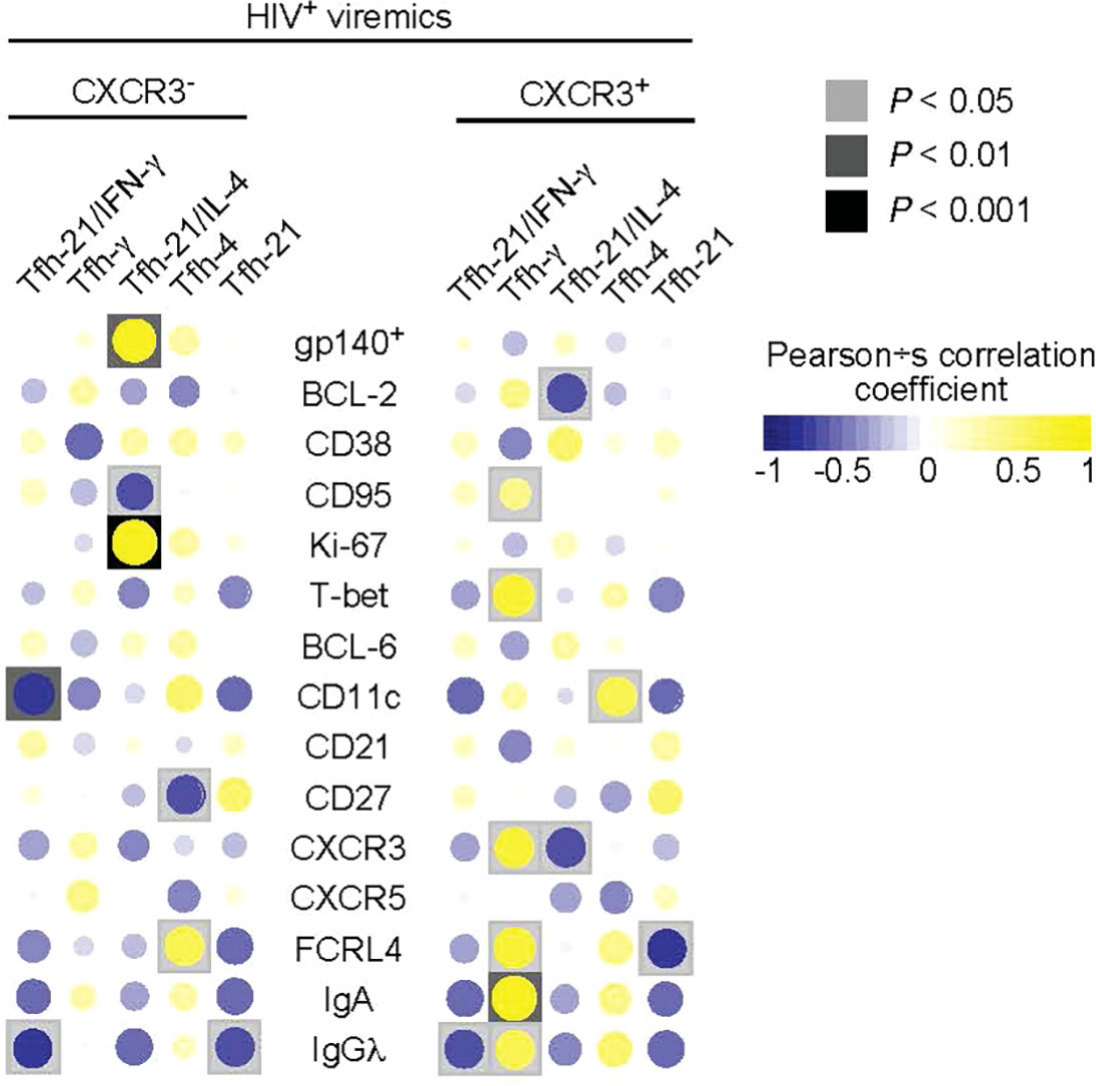
Correlations between cytokines producing Tfh cells and frequency and phenotype of gp140 specific B cells. Correlative heat maps between the frequency of cytokines produced by Tfh cells from HIV infected viremic individuals and the proportion of gp140 specific B cells and their phenotype (N = 11). Correlative analyses were performed on log10 transformed frequencies using Pearson’s test.

Having defined phenotypically and functionally distinct Tfh cell populations and the phenotype of LN gp140-specific B cells associated with ongoing HIV replication, we next analyzed the relationship between the functional profile of Tfh cells and the phenotype and function of gp140-specific B cells. As shown above, the CXCR3^-^ Tfh cell population consists of single IL-21, dual IL-21/IL-4 and single IL-4 producing Tfh cells thus defining a Th2-like Tfh cell population while the CXCR3^+^ Tfh cell population defines a Th1-like Tfh cell population containing dual IL-21/IFN-γ and single IFN-γ Tfh cells (**Figure 3C**). The correlation heat map in **Figure 5** shows that the frequency of gp140^+^ B cells in HIV-infected viremic individuals was positively correlated with the proportion of dual IL-21/IL-4 CXCR3^-^ Th2-like Tfh cells (*r* = 0.82, *P* = 0.001). Similarly, the proportion of dual IL-21/IL-4 CXCR3^-^ Th2-like Tfh cells was negatively correlated with the frequency of CD95^+^gp140^+^ B cells (*r* = -0.61, *P* = 0.04) and was positively correlated with the frequency of Ki-67^+^gp140^+^ B cells (*r* = 0.87, *P* = 0.0004) suggesting that IL-4 in combination with IL-21 drives the expansion of HIV specific B cells. However, the frequency of single IFN-γ/IL-21CXCR3+ and IFN-γ CXCR3^+^ Th1-like Tfh cells in HIV-infected viremic individuals did not correlate with the percentage of gp140-specific B cells and was positively correlated with increased expression of T-bet (*r* = 0.71, *P* = 0.014), CXCR3 (*r* = 0.7, *P* = 0.015) and FCRL4 (*r* = 0.71, *P* = 0.013) on gp140^+^ B cells, a polulation of LN B cells that has been previously associated with poor affinity maturation and poor immunologic outcome (28, 29).

Having identified distinct signatures in both the Tfh and B cell compartments in HIV-1 infected individuals, we sought to provide formal demonstration of the influence of the Th1-like Tfh and Th2-like Tfh cells on modulating the function and phenotype of B cells.

We evaluated the effects of cytokine treatment with IL-21, IL-4 and IFN-γ on the induction of the expression of CXCR3 and T-bet in different memory B cell populations from tonsils of HIV negative pediatric donors. Tonsil cells were used due to the large number of tissue B cells necessary to perform these experiments. IFN-γ consistently induced expression of CXCR3 and T-bet in unswitched, switched and GC B cell populations (**Supplementary Figure 5**). IL-21 and IL-4 showed no effects, with the exception of IL-4 inducing T-bet expression in unswitched memory B cells. R848, a strong agonist of TLR7/TLR8, was used as positive control and showed an effect similar to IFN-γ.

**Figure 6:**
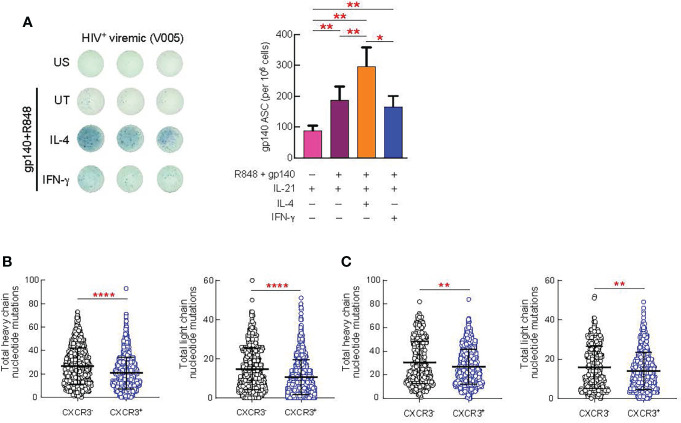
Effect of *in vitro* cytokine stimulation of B cells on antibody production and SHM. Lymph node (LN) mononuclear cells from 9 HIV^+^ viremic individuals were stimulated or not for 4 days presence of gp140 (0.1 ug/ml) and R848 and in prensence or absence of IL-4 (100 ng/ml) and IFN-γ (100 ng/ml). **(A)** Frequencies of gp140-specific antibody secreting cells (ASC) were measured by ELISPOT. The left panel shows representative counting of spot-forming cells (SFC) and the right panel shows the frequency of gp140 ASC calculated from triplicate wells plated with 100.000 LNMCs per well. Total somatic mutations per antibody were determined by paired chain sequencing of CXCR3^+^ and CXCR3^-^ IgG B cells **(B)** or CXCR3^+^ and CXCR3^-^ gp140-specific B cells **(C)** from five donors. Error bars correspond to mean ± SEM and statistical significance was evaluated using Wilcoxon signed-rank test and in **(D, E)** by Mann-Whitney test. P values *P < 0.05, **P < 0.01, ****P < 0.0001.

Next, we investigated the impact of IL-4 and IFN-γ treatment on the maturation of HIV-specific B cell responses from 9 HIV viremic individuals after 4 days of stimulation in the presence of gp140 protein, IL-21 and suboptimal doses of R848. As shown in **Figure 6A**, HIV-specific B cell responses from unstimulated LNMC, as measured by ELISPOT, were low but detectable and their frequencies significantly increased in all the conditions when cells were stimulated with gp140+R848 (P<0.01). Interestingly, IL-4 stimulation led to a significant increase in the proportion of gp140 ASC cells when compared to gp140+R848 stimulated cells and R848+IFN-γ stimulation, 1.5 fold, (*P*=0.0039) and 1.8 fold (P=0.01), respectively (**Figure 6A**). Of note, the frequencies of HIV-specific B cells after IFN-γ stimulation were not significantly different from those observed with gp140+R848 stimulation alone.

Finally, we determined whether there were differences in the quality of the antibodies produced by CXCR3^+^ versus CXCR3^-^ GC B cell populations. The rationale for these experiments is supported by our observation that Th1-like Tfh cells drive the expansion of the CXCR3^+^T-bet^+^ GC B cells. Furthermore, consistent with previous studies (29, 30) we observed an increased frequency of T-bet^+^CXCR3^+^ B cells in the lymph nodes of HIV-infected viremic individuals as compared to HIV uninfected and HIV-infected ART treated individuals individuals (*P* < 0.0001) (**Supplementary Figure 6A**), and all T-bet^+^ B cells were contained within the CXCR3^+^ B cell population (*P* < 0.0001) (**Supplementary Figure 6B**). Therefore, we isolated CXCR3^+^ and CXCR3^-^ IgG^+^ B cells from five viremic individuals and assessed levels of somatic hypermutation (SHM) by carrying out error-corrected sequence analysis of natively paired heavy and light chain genes for LN B cells. We found that the level of SHM differed by B cell CXCR3 status (**Figures 6B, C**). On average, the CXCR3^+^ phenotype was associated with significantly lower levels of SHM than the CXCR3^-^ phenotype (*P* = 0.0003 for the heavy chain and *P* < 0.0001 for the light chain) (**Figure 6B**). Along the same line, the level of SHM was significantly lower in gp140-specific CXCR3^+^ as compared to CXCR3^-^ B cells (*P* = 0.0025 for the heavy chain and *P* = 0.0088 for the light chain) (**Figure 6C**).

Taken together these results indicate that the skewed Th1-like Tfh versus Th2-like Tfh cells cytokine profiles associated with active HIV replication influence the phenotype, the maturation, the magnitude and the quality of HIV-specific B cell responses.

## Discussion

In the present study, we have studied lymph nodes from three study groups including HIV-uninfected, HIV-infected ART treated and untreated viremic individuals to delineate the differences in phenotypic and functional profiles and to determine whether the different profiles influence the development of B cell responses. While, a series of studies (31–33) demonstrated that HIV replication was associated with progressive damages to the microarchitecture of LNs, that consequently may influence T and B cell phenotype and functions, the influence of duration of HIV infection was not assessed in the present study and would therefore require further investigations.

The unsupervised approach used to analyse distributions of thirty markers of T cell activation, memory differentiation, chemokine receptors and HIV coreceptors has allowed us to identify a number of phenotypic markers defining differences between the three study groups. CXCR3 and CD38 contributed most to the heterogeneity of Tfh cells between the three study groups. Phenotypic and functional diversity has also previously been shown within different Tfh cell populations from tonsils and HIV-infected viremic individuals using mass cytometry (16, 21, 34).

The Tfh cell population greatly expanded in HIV-infected viremic individuals was characterized by the co-expression of CXCR3 and CD38 while Tfh cells lacking both markers were largely (about 60%) represented in HIV negative subjects. Interestingly, the expression or the lack of CXCR3 distinguishes between Th1-like Tfh cells expressing T-bet and Th2-like Tfh cells expressing GATA-3, respectively. These results are consistent with the conclusions from other studies pointing out an increased activation of Tfh cells and a higher proportion of CXCR3^+^ Tfh cells in HIV-infected viremic infection (13, 16, 30, 35). However, we demonstrate that CXCR3 is the phenotypic marker distinguishing between Th1-like and Th2 like Tfh cells both in HIV-uninfected and HIV infected individuals.

An additional important observation of our study is the functional dichotomy between CXCR3^+^ and CXCR3^-^ Tfh cells in the secretion of Th1 (IFN-γ) and Th2 (IL-4) cytokines. Previous studies performed in mice have shown the critical role of IL-21 in driving the expansion of GC B cells (36–43). However, IL-21 is not sufficient for the optimal maturation of the GC response, which also requires Tfh cells producing IL-4, which seem to further regulate the migration of GC B cells between the dark and light zones (11). CXCR3^+^ Tfh cells mainly produce dual IL-21/IFN-γ and single IFN-γ cytokines while CXCR3^-^ Tfh cells mainly produce single IL-21, dual IL-21/IL-4 and single IL-4 cells. We have not observed a defect in IL-21 producing Tfh cells, which is consistent with the significant expansion of GC B cells (both total and HIV-specific) associated with active HIV replication. However, we have observed a selective reduction, about a 4 fold reduction, in the total percentage of Tfh cells producing IL-4 (dual IL-21/IL-4 + single IL-4) between HIV-uninfected and viremic individuals, thus suggesting a potential defect in the maturation of the B cell response in viremic individuals. Of note, ART was not able to significantly modulate the Th2-like Tfh population.

We then further characterized the phenotype and the maturation of HIV-specific B cells as compared to B cells specific to Flu isolated from lymph nodes of the same individuals. HIV-specifc B cells were differently distributed as compared to Flu-specific, being the former mostly contained within GC B cells and the latter in the switch memory B cells. HIV-specific B cells from HIV-infected viremic individuals showed significant higher percentage of cycling cells and an activated phenotype and also a pro-apoptotic profile as indicated by the reduced expression of BCL-2 and increased expression of CD95. These observations further support previous studies^12,14^ indicating that B cell expansion during actively replicating HIV infection is driven by HIV. However, when lymph node Flu-specific B cells from HIV-infected viremic individuals were compared to those of HIV-uninfected and HIV-infected ART treated individuals, they also showed significant increase in markers of activation and maturation, an apoptotic phenotypic profile and increase expression of T-bet. Therefore, despite the absence of Flu-specific stimulation these results suggest that the Th1 cytokine microenviroment associated with viremic HIV infection may be responsible for the changes in maturation, activation and phenotype of Flu-specific B cells from viremic as compared to HIV-uninfected individuals.

We then determined the influence of the cytokine microenvironment and, in particular, of the Th1/Th2 cytokine imbalance on the phenotype and the optimal maturation of the B cell response in individuals with active replicating HIV infection. Our results indicate that IFN-γ induces the expression of T-bet and CXCR3, observed in GC B cells of viremic individuals. The additional changes in the phenotype of gp140-specific B cells such as the increased in the CD11c and FCRL-4 expression are in line with previous studies indicating that these phenotypic abnormalities are associated with chronic stimulation (27, 29, 44–46). Furthermore, the positive correlation of the percentage of single IFN-γ Th1-like Tfh cells with T-bet^+^, CXCR3^+^, CD95^+^ and FCRL4^+^ HIV-specific B cells further support the results obtained *in vitro* and also indicates that HIV-specific B cells induced by Th1-like Tfh cells are potentially prone to apoptosis and limited proliferation. In contrast, dual IL-21/IL-4 Th2-like Tfh cells cells were positively correlated with the percentage of total and dividing (Ki-67^+^) HIV-specific B cells and negatively correlated with the expression of CD95 suggesting that HIV replication may shape the phenotypic profile of HIV-specific B cells.

Moreover, a reconstructive *in vitro* experiment showed that IL-4 but not IFN-γ in combination with IL-21 increased the maturation and cycling of LN gp140-specifc B cells. In contrast, *in vitro* IFN-γ stimulation induced the differentiation of gp140-specific B cells expressing T-bet^+^ B cells that showed significantly lower levels of somatic hypermutation as compared to CXCR3^-^ cells. These data indicated that the imbalance in the Th1 versus Th2-like Tfh cytokine profile probably affects the maturation of B cell response.

Our results therefore support the model that the defect in Th2-like/Tfh cells in favor of Th1-like Tfh cells is an important mechanism to explain the unique phenotypic profile of B cells and the impaired maturation of B cell response in viremic individuals. These results support the development of optimal Th2-like Tfh cell responses in HIV immunization strategies aiming at the development of quantitatively and qualitatively effective antibody responses.

## Materials and methods

### Experimental design

Lymph node biopsies were performed in 24 HIV-1 infected viremic individuals naive to antiretroviral therapy and 29 HIV-infected ART treated individuals subjects (**Table 1**). With regard to HIV negative subjects, lymph node biopsies (inguinal lymph nodes) were performed in 18 subjects who underwent vascular (varicose vein stripping) and general (uncomplicated bilateral inguinal herniorrhaphy) surgery. Tonsils were obtained from young patients who underwent tonsillectomy. These studies were approved by the Institutional Review Board of the Centre Hospitalier Universitaire Vaudois, and all subjects gave written informed consent. For all the experiments, participant ID were randomized, and the samples were randomly numbered to perform the experiments. No outliers were excluded from the analyses.

### Isolation of lymph node and tonsil mononuclear cells

Lymph node and tonsil mononuclear cells were isolated by mechanical disruption as previously described (47) and cells were cryopreserved in liquid nitrogen.

### CyTOF marker labeling and detection

Cryopreserved lymph node mononuclear cells (LNMCs) were thawed and resuspended in complete RPMI medium (Gibco; Life Technologies; 10% heat-inactivated FBS [Institut de Biotechnologies Jacques Boy], 100 IU/ml penicillin, and 100 µg/ml streptomycin [BioConcept]). For the T cell panel 2x10^6^ cells/ml were stimulated or not with 100 ng/ml PMA (Sigma-Aldrich) and 1 µg/ml ionomycin (Sigma-Aldrich) in the presence of golgi plug (BD) for 5 hours at 37°C.

For the B cell panel cells were blocked using unlabeled anti-CD4 pure (clone SK3, BD Bioscience) antibody as previously described (48). Cells were washed twice and then incubated for 30 minutes at 4°C with gp140 (Consensus B) biotinylated bound to a streptavidin PE. Two biotinylated flu probes bound to APC were used as previously shown (49): one from H1 strain CA09 (for samples collected during or after the 2009-2010 season) and one from NC-99 (for samples collected prior to the 2009-2010 season).

Viability of cells in 500 μl of PBS was identified by incubation with 50 μM cisplatin (Sigma-Aldrich) for 5 min at RT and quenched with 500 μl fetal bovine serum. Next, cells were incubated for 30 min at 4°C with a 50 μl cocktail of cell surface metal conjugated antibodies (Fluidigm/DVS Science). Cells were washed and fixed for 10 min at RT with 2.4% PFA. Next, cells were permeabilized for 45 min at 4°C with Foxp3 Fixation/Permeabilization kit (eBioscience), washed and stained at 4°C for 30 min with a 50 μl cocktail of transcription factor and cytokine metal conjugated antibodies. Cells were washed and fixed for 10 min at RT with 2.4% PFA. Total cells were identified by DNA intercalation (1 μM Cell-ID Intercalator, Fluidigm/DVS Science) in 2% PFA at 4°C overnight. The list of metal isotopes antibodies used are listed in **Tables S1A, B**. Labeled samples were assessed by the CyTOF1 instrument that was upgraded to CyTOF2 (Fluidigm) using a flow rate of 0.045 ml/min.

### CyTOF data analysis

FCS files were normalized to the EQ Four Element Calibration Beads using the CyTOF software. For conventional cytometric analysis of B and Tfh cell populations, FCS files were imported into Cytobank Data Analysis Software or FlowJo v10.4.2 (Treestar, Inc., Ashland, CR) and SPICE v5.3 (developed by Mario Roederer, National Institute of Health) (50). Gated Tfh cells were imported into R software using the flowWorkspace framework (51). Marker intensity values were arcsinh (hyperbolic inverse sine) with cofactor 5 transformed. Unsupervised clustering was conducted using FlowSOM (52) (BuiltSOM function in FlowSOM package) on pooled Tfh cells from all samples (92’116 cells) (in combination with hierarchical consensus meta-clustering (metaClustering_consensus function in FlowSOM package). Dimensionality reduction was performed using the Barnes-Hut implementation of t-distributed stochastic neighbor embedding (Rtsne function in Rtsne package).

Principal component analysis was performed on single cell data and the absolute values of the marker loadings of the first two principal components were averaged and reported in **Figure 1F**.

### Elispot assay

LNMCs were stimulated or not for 4 days with 0.1 ug/ml of gp140 and 1 ug/ml of R848 (*In vivo*Gen), 10 ng/ml of IL-2 (Miltenyi Biotec) and 100 ng/ml of IL-21 (Miltenyi Biotec). In the stimulated conditions cells were treated or not with 100 ng/ml of IL-4 (Miltenyi Biotec) or IFN-γ (Miltenyi Biotec). ELISPOT plates (BD) were coated with 15 ug/ml of anti-Ig antibodies (Mabtech) at 4°C overnight. Next, plates were washed and cells were added for 24 hours at 37°C followed by addition of biotinylated antibody against IgG or biotinylated proteins gp140 or the control protein keyhole limpet hemocyanin (KLH), and finally addition of a streptavidin-HRP (Mabtech). Frequencies of gp140-specific antibody secreting cells (ASC) were calculated from triplicate wells plated with 100.000 LNMCs per well. Specificity was verified with PBMCs of HIV-uninfected individuals.

### Paired chain antibody sequencing

Single CD19^+^CD20^+^CD3^-^CD14^-^IgA^-^IgM^-^IgD^-^ IgG^+^ cells and gp140^+^ B cell were sorted on the basis of CXCR3 expression into wells of 384-well plates by FACS. Generation of barcoded cDNA, PCR amplification, and sequencing of IgG genes were performed as described in Tan et al., 2014 (53), with the following modifications: biotinylated Oligo(dT) and RT maxima H- (Fisher Scientific Company) were used for reverse transcription, cDNA was extracted using Streptavidin C1 beads (Life Technologies), and DNA concentrations were determined using qPCR (KAPA SYBR^®^ FAST qPCR Kit for Titanium, Kapabiosystems). V(D)J assignment and mutation identification was performed using a variant of SoDA (54).

### Statistical analysis

GraphPad PRISM and R softwares were used to perform statistical analyses.

Linear regressions were performed to compare frequencies (log10 transformed) of Tfh clusters or antigen specific B cells among the different study groups and the resulting *P* values were adjusted for multiple testing using the Benjamini-Hochberg FDR method (with significance cutoff set at 0.05).

Statistical analyses comparing cell surface markers, transcription factors and cytokine production (log10 transformed) in Tfh subsets defined by CD38 and CXCR3 were assessed by linear mixed-effect models accounting for differences between patient groups (HIV-uninfected, HIV infected ART-treated and viremic individuals) with patient-level random intercepts. *P* values were adjusted using the Benjamini-Hochberg FDR method (with significance cutoff set at 0.05).

Two-tailed Mann-Whitney unpaired test was used to compare frequencies of B cell subsets between the three different groups (HIV-uninfetced, HIV-infected ART treated and viremic individuals).

Correlative analyses were performed on log10 transformed frequencies using Pearson’s test. The Wilcoxon signed-rank paired test was used to detect differences between variables from the same sample.

## Data availability statement

The original contributions presented in the study are included in the article/**Supplementary Material**. Further inquiries can be directed to the corresponding author.

## Ethics statement

The studies involving human participants were reviewed and approved by La Commission cantonale d’éthique de la recherche sur l’être humain du Canton de Vaud. The patients/participants provided their written informed consent to participate in this study.

## Author contributions

AN designed and performed the experiments, analyzed the data, and wrote the manuscript. MS performed data analyses. VJ and MP performed mass cytometry stainings. FP and AM performed the HIV RNA quantification. GC and YL performed the Paired Chain Antibody Sequencing. J-MC performed lymph node biopsy. MC and AR recruited participants. LS, ABM and RK provided gp140 and HA probes and helped with experimental design. RG and CF and MP provided help in mass cytometry data analysis. GP desined the overall study, provided conceptual advice and wrote the manuscript. All authors contributed to the article and approved the submitted version.

## Funding

This work was supported by Swiss National Science Foundation Grant 320030_200912 and by Freedom Forever Association to MP and by grants of Bill and Melinda Gates Foundation OPP_1114725 to GP. The funders had no role in the study design, data collection and interpretation, or the decision to submit the work for publication.

## Acknowledgments

We are grateful to Hottinger Rosemary, the study manager and to Line Esteves-Leuenberger, Alex Farina, Thibaut Decaillon, Manon Geiser and Michael Moulin for technical assistance.

## Conflict of interest

GC and YL were employed by the company Atreca, Inc.

The remaining authors declare that the research was conducted in the absence of any commercial or financial relationships that could be construed as a potential conflict of interest.

## Publisher’s note

All claims expressed in this article are solely those of the authors and do not necessarily represent those of their affiliated organizations, or those of the publisher, the editors and the reviewers. Any product that may be evaluated in this article, or claim that may be made by its manufacturer, is not guaranteed or endorsed by the publisher.
