# Supplementary material for: The deficiency in Th2-like Tfh cells affects the maturation and quality of HIV-specific B cell response in viremic infection
Source: Front Immunol. 2022 Aug 24;13:960120. doi: 10.3389/fimmu.2022.960120 (PMC9450063; doi:10.3389/fimmu.2022.960120)
Supplement: Supplementary file 1 [file DataSheet_1.pdf]

Supplementary Figure 1

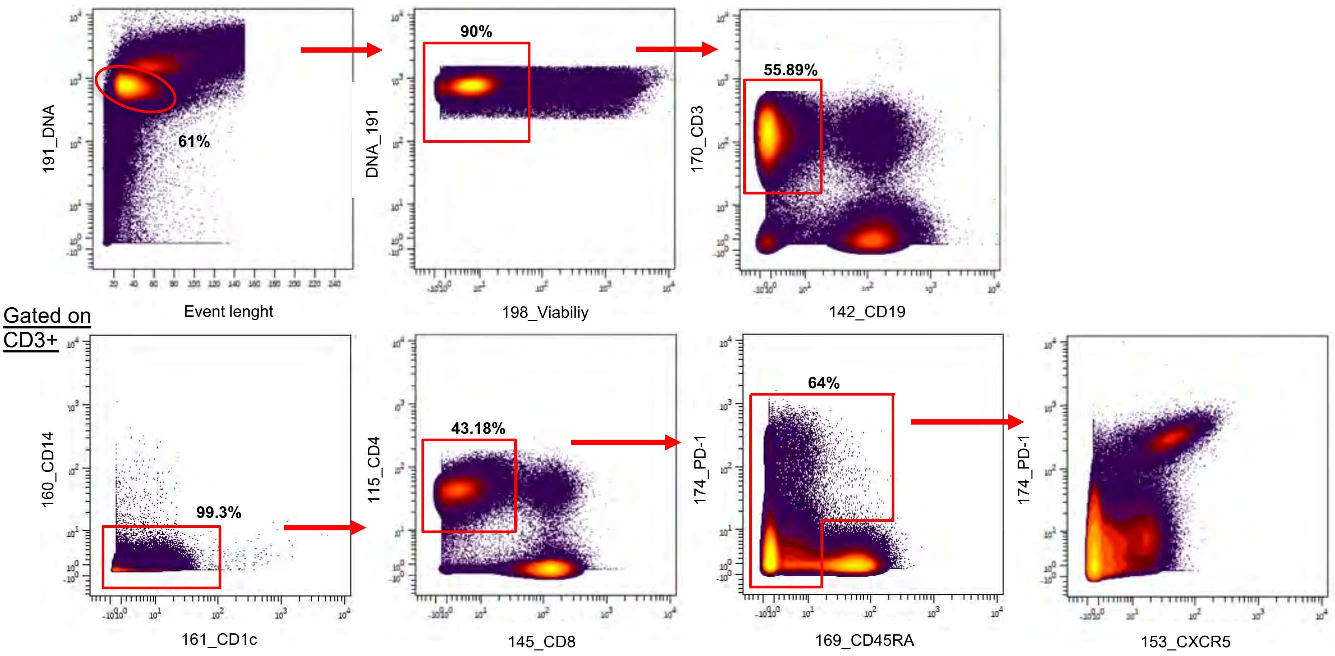

Supplementary Figure 1: Mass cytometry gating strategy to define Tfh cells.

Supplementary Figure 2

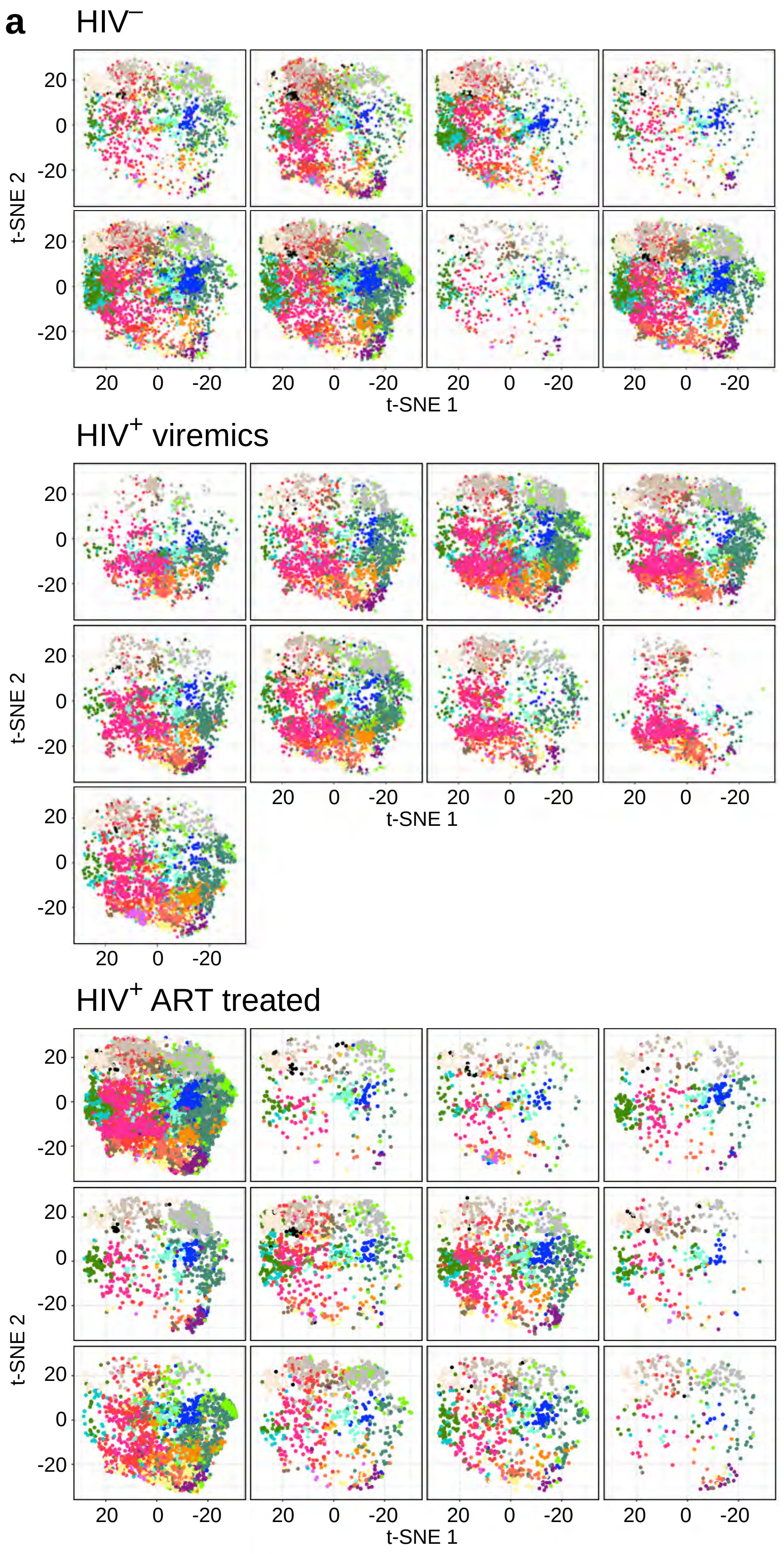

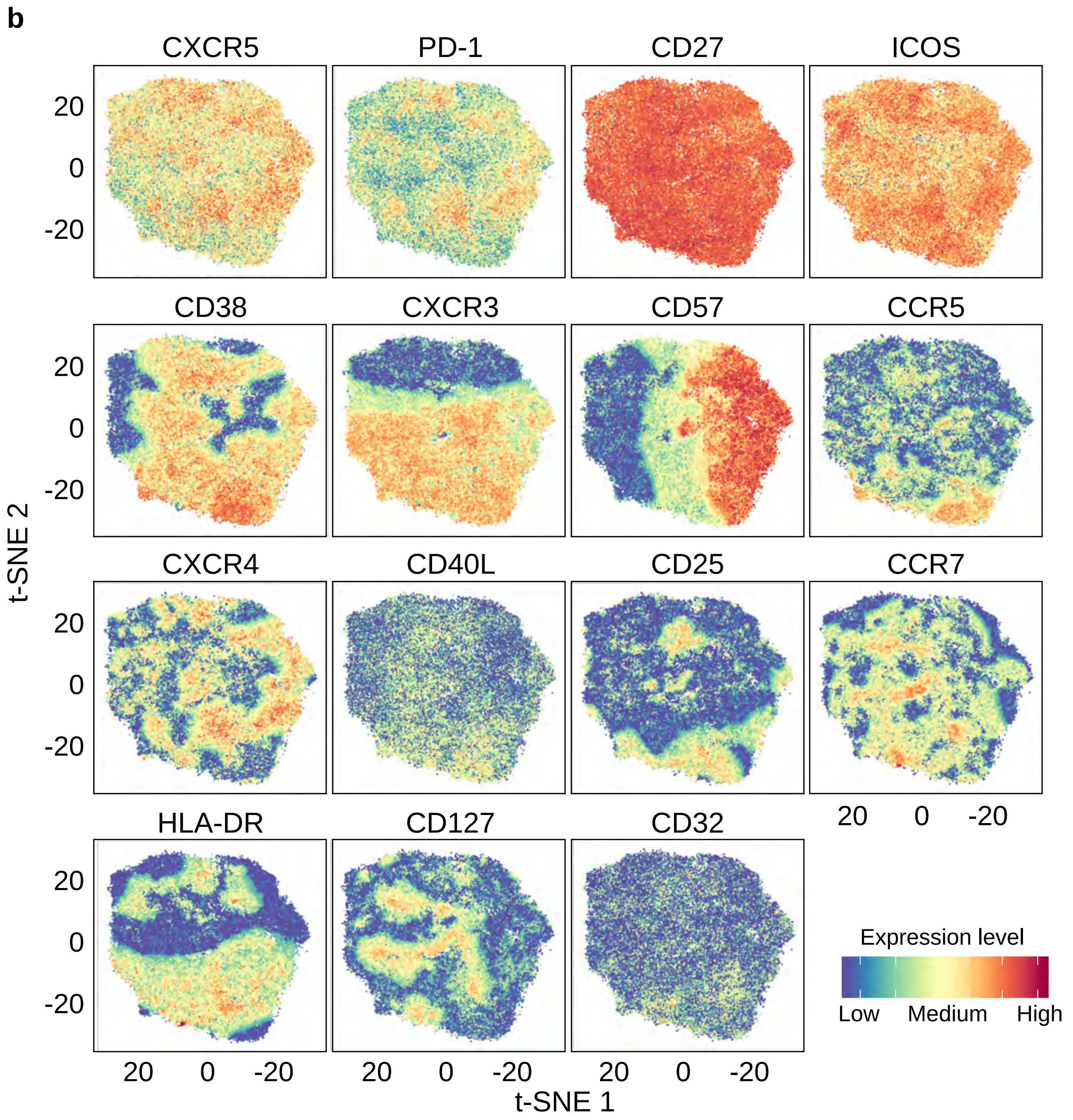
b
CXCR5
PD-1
CD27
ICOS
CD38
CXCR3
CD57
CCR5
CXCR4
CD40L
CD25
CCR7
HLA-DR
CD127
CD32
t-SNE 2
t-SNE 1
20
0
-20
Expression level
Low
Medium
High

c

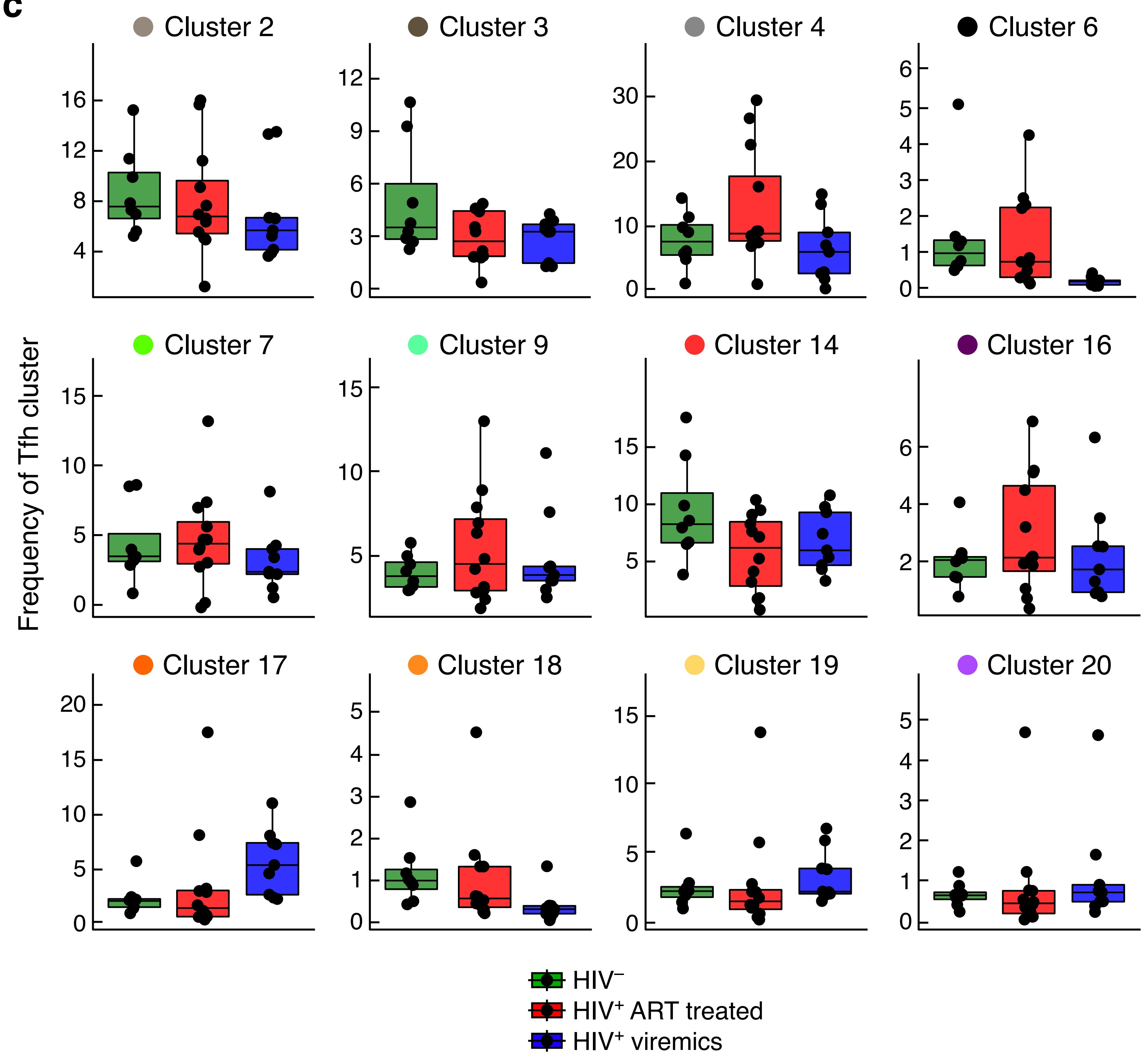

Supplementary Figure 2: High-dimensional analysis of Tfh cells by mass cytometry.
a) Individual t-SNE plots of each HIV-uninfected (N = 8), HIV-infected ART treated (N = 12) and viremic individuals (N = 9). b) Signal intensity of individual markers on t-SNE plots. Tfh cells were pooled (N = 92'162 cells) and colored according to the scaled expression level of indicated markers. c) Tfh clusters not significantly different between the three study groups. Linear regressions were performed to compare frequencies of Tfh clusters across the three groups (HIV- (green), HIV-infected ART treated (red) and viremic (blue) individuals.

Supplementary Figure 3

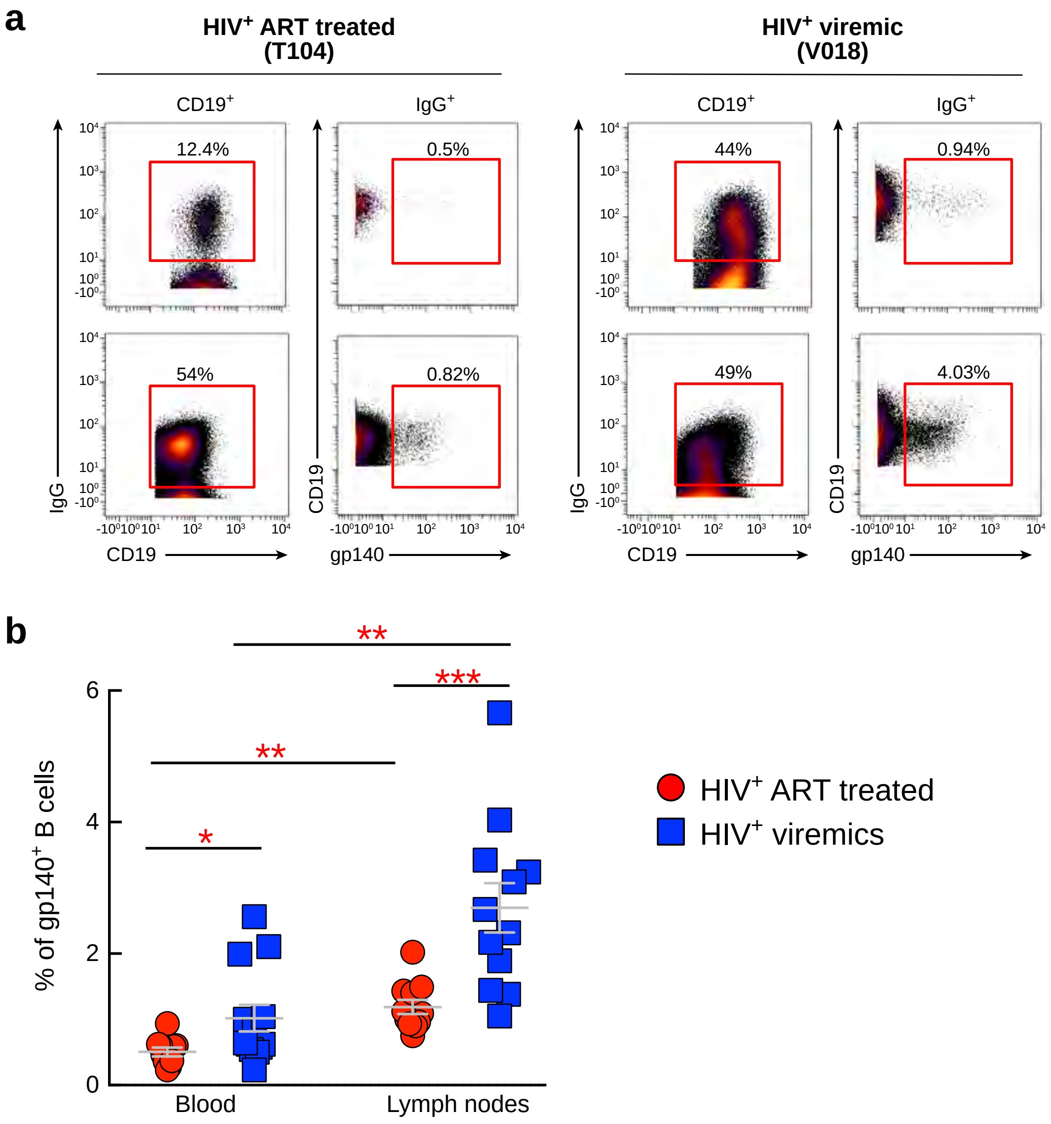

Supplementary Figure 3: Frequency of gp140 specific B cells from HIV-infected ART treated and viremic individuals in blood versus lymph nodes. a) Representative mass cytometry profiles of blood and LN CD19+ IgG+ B cell populations binding to gp140 probes in representative HIV-infected ART treated individuals and viremic subjects. b) Cumulative data on the frequencies of gp140-specific B cells in blood and LN mononuclear cells of HIV-infected ART treated individuals (red) and viremic (blue) individuals. Statistical significance (P values) were calculated using Mann-Whitney test to compare the two groups and a Wilcoxon signed-rank test to compare frequencies between blood and LNs. * $P < 0.05$, ** $P < 0.01$, *** $P < 0.001$. Error bars denote mean ± S.E.M.

Supplementary Figure 4

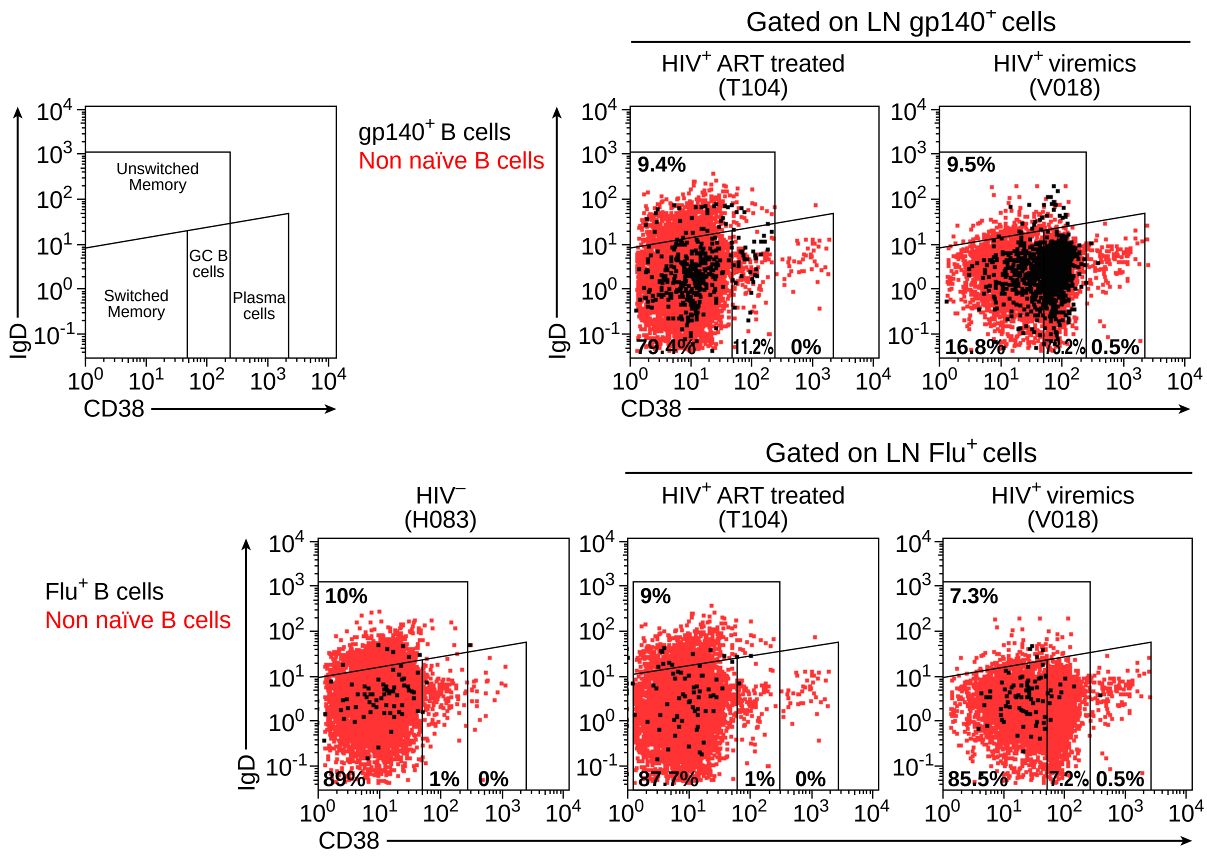

Supplementary Figure 4: Representative mass cytometry profile of gp140 and flu specific B cells in LN B cell populations defined by IgD and CD38 expression. gp140+ B and Flu+ B cells (black dots) from representative HIV-, HIV-infected ART treated individuals and viremic individuals by mass cytometry. Gates were set using total memory B cells (red dots).

Supplementary Figure 5

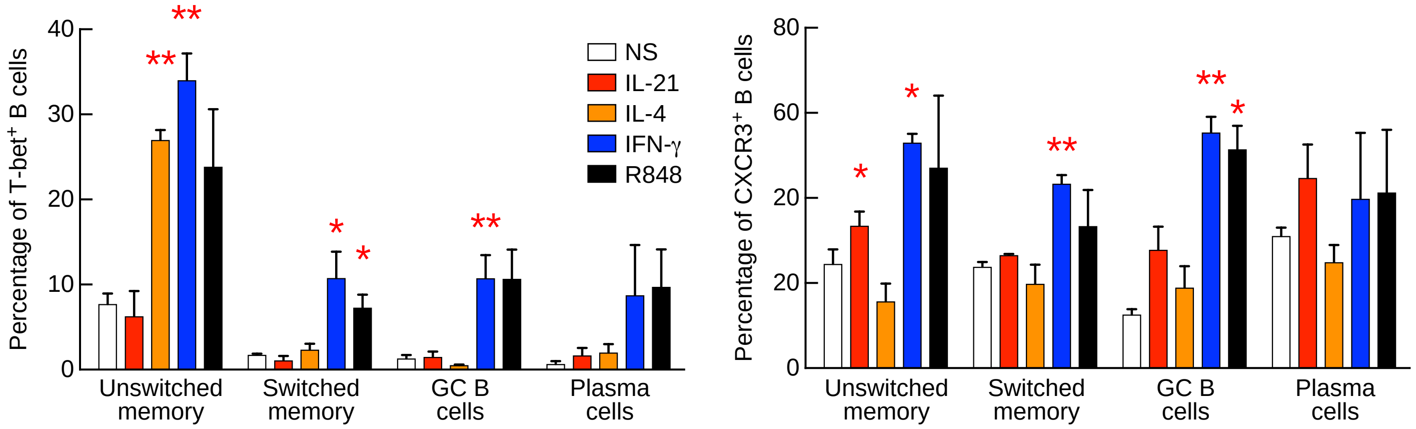

Supplementary Figure 5. Effect of in vitro cytokine stimulation of B cells. Tonsil (TN) mononuclear cells were cultured for 3 days in the presence or absence of IL-21 (100 ng/ml), IL-4 (100 ng/ml), IFN-γ (100 ng/ml) and R848 (1 μg/ml). Mass cytometry staining was performed using anti-CD19, anti-CD27, anti-CD38, anti-IgD, anti-CXCR3, and anti-T-bet antibodies (N = 3). Percentage of T-bet+ and CXCR3+ B cells after 3 days of culture.

Supplementary Figure 6

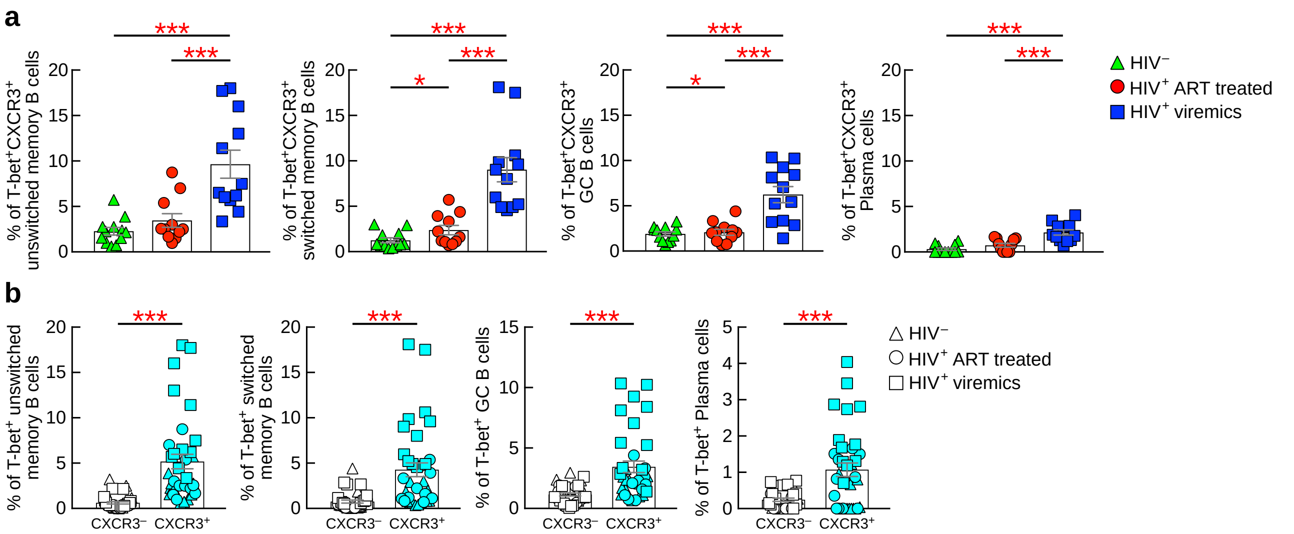

Supplementary Figure 6. Frequency of T-bet+ CXCR3+ B cells subsets from HIV uninfected, HIV-infected ART treated and viremic individuals. Non naive B cells were gated on unswitched memory (IgD+CD38-), switched memory (IgD-CD38-), GC (IgD-CD38+) and plasma cells (IgD-CD38hi) B cell populations and (a) the percentage of T-bet+ CXCR3+ B cells was analyzed by mass cytometry. (b) Percentage of T-bet+ B cells within the CXCR3+ and CXCR3- B cells. In (a) statistical significance (P values) were calculated using Mann-Whitney test while in (b) by Wilcoxon signed-rank test * P < 0.05, ** P < 0.01, *** P < 0.001. Error bars denote mean ± S.E.M.

Table S1

a **Mass cytometry T cell panel.**

Target	Metal	Company	Clone
CD4	115In	Biolegend	RPA-T4
CCR6	141Pr	Fluidigm/DVS	G034E3
CD19	142Nd	Fluidigm/DVS	HIB19
ICOS	143Nd	Biolegend	C398.4A
CD8	145Nd	Biolegend	RPA-T8
IgD	146Nd	Fluidigm/DVS	IA6-2
CD7	147Sm	Fluidigm/DVS	CD7-6B7
CD57	148Nd	BD	G10F5
CCR4	149Sm	Fluidigm/DVS	205410
CXCR3	153Eu	Fluidigm/DVS	RF8B2
CD21	152Sm	Fluidigm/DVS	BL13
CXCR3	154Sm	BIolegend	G025H7
CD27	155Gd	Fluidigm/DVS	L128
CD11c	156Gd	Biolegend	3.9
CCR7	159Tb	Fluidigm/DVS	G043H7
CD25	158Gd	Biolegend	M-A251
CD14	160Gd	Fluidigm/DVS	M5E2
CD1C	161Dy	Biolegend	L161
CD32-APC	162Dy	Fluidigm/DVS	FUN2
CD20	166Er	Biolegend	2H7
CD38	167Er	Fluidigm/DVS	HIT2
CD45RA	169Tm	Fluidigm/DVS	HI100
CD40L	168Er	Fluidigm/DVS	CD40L
CD3	170Er	Fluidigm/DVS	UCHT1
CCR5	171Yb	Fluidigm/DVS	NP-6G4
HLA-DR	173Yb	Fluidigm/DVS	L243
PD-1	174Yb	Fluidigm/DVS	EH12.2H7
CXCR4	175Lu	Fluidigm/DVS	12G5
CD127	176Yb	Fluidigm/DVS	A019D5
CD16	209BI	Fluidigm/DVS	3G8
Live/Dead	195Pt	Fluidigm/DVS	Cell-ID

b Mass cytometry B cell panel.

Target	Metal	Company	Clone
IgG*	113-In	BD	G18-145
Blimp-1	115-In	BIO-TECHNE AG	646702
PARP	141-Pr	BD	F21-852
CD19	142-Nd	DVS	HIB19
HLADR*	143-Nd	DVS	L243
CD38*	144-Nd	DVS	HIT2
CD8	145-Nd	Biolegend	RPA-T8
IgD*	146-Nd	DVS	IA6-2
CD20*	147-Sm	DVS	2H7
IgA*	148-Sm	DVS	Polyclonal
CD79A	149-Sm	Biolegend	gα (alpha)
CD138	150-Nd	DVS	DL-101
IgG lambda*	151-Eu	DVS	MHL-38
CD21*	152-Sm	DVS	BL13
CXCR5*	153-Eu	DVS	RF8B2
CXCR3*	154-Sm	Biolegend	G025H7
CD27*	155-Gd	DVS	L128
gp140-PE	156-Gd	DVS	PE001
ICOS-L	158-Gd	Biolegend	2D3
CD11C*	159-Tb	DVS	Bu15
IgG kappa*	160-Gd	DVS	MHK-49
T-bet*	161-Dy	DVS	4B10
H1N1-APC	162-Dy	DVS	APC003
BCL6*	163-Dy	DVS	K112-91
CD95*	164-Dy	DVS	FAS
CD40*	165-Ho	DVS	5C3
CD24*	166-Er	DVS	ML5
FCRL4*	167-Er	Biolegend	413D12
Ki-67*	168-Er	DVS	Ki-67
CD45RA*	169-Tm	DVS	HI100
CD3	170-Er	DVS	UCHT1
CD83*	171-Yb	Biolegend	HB15e
IgM*	172-Yb	DVS	MHM-88
BCL-2*	173-Yb	Biolegend	100
PD-1	174-Yb	DVS	EH12.2H7
CXCR4*	175-Lu	DVS	12G5
CD4	176-Yb	DVS	RPA-T4

*used for MDS analysis
